# Renal cell carcinoma presenting as a tumor on the scalp: A case report

**DOI:** 10.1016/j.ijscr.2020.09.122

**Published:** 2020-09-24

**Authors:** Christina Krogerus, Matilda Svenning, Anette Pedersen Pilt, Hannah Trøstrup

**Affiliations:** a Department of Plastic Surgery and Breast Surgery, Zealand University Hospital, Roskilde, Denmark; b Department of Pathology, Zealand University Hospital, Roskilde, Denmark

**Keywords:** Renal cell carcinoma, Skin metastasis, Case report

## Abstract

**Introduction:**

Renal cell carcinoma (RCC) is often diagnosed as an incidental finding on imaging studies and about a fourth of patients have metastases by the time of diagnosis. RCC is known to metastasize widely but cutaneous metastases are considered uncommon and are rarely the presenting symptom of RCC. We present a case of RCC presenting with a tumor on the scalp.

**Case:**

A 65-year-old healthy man presented with a 9-month history of a growing mass on the scalp. Clinical examination revealed a pulsating, highly vascularized tumor that was excised. Histopathological findings were consistent with a metastasis from a renal cell carcinoma. Further investigations revealed a 13 cm kidney tumor with metastases to the lungs and mediastinum.

**Discussion:**

Cutaneous metastasis from RCC is uncommon and is rarely the initial symptom of RCC. The lesions often have a vascularized appearance and may be clinically confused with other vascular tumors. Skin metastasis is a sign of advanced disease and prognosis is poor.

**Conclusion:**

An occult RCC may present with a skin lesion. Skin metastasis from RCC is an important differential diagnosis in tumors with a vascular appearance, especially in patients with a previous history of RCC.

## Highlights

• Skin metastases from renal cell carcinomas are rare.
• Skin metastases might have a vascularized appearance.
• Metastasis to the skin is associated with a poor prognosis.

## 1 Introduction

Renal cell carcinomas (RCC) account for approximately 2% of adult malignancies. It is often diagnosed as an incidental finding during imaging and the classic triad of haematuria, flank pain and palpable mass only occurs in about 10% of patients [1]. RCC is known to metastasize widely and in 25–30% of cases the cancer has metastasized by the time of diagnosis [2]. The most common locations for metastasis are lungs, liver and bone. Metastases to the skin are unusual and is considered a late manifestation of the disease. Thus, skin metastases are rarely seen as the initial presentation of RCC and are associated with an unfavorable prognosis. We present a case of a patient, where a growing tumor on the scalp was the first symptom of a silent renal cell carcinoma. This work has been reported in line with the SCARE criteria [3].

## 2 Case

A 65-year-old healthy, non-smoking, male was referred to the plastic surgery department due to a growing mass on the scalp. The patient had first noticed the mass about 9 months earlier. He did not have any physical discomfort related to it but became worried because of the increasingly rapid growth. The patient did not report any symptoms of weight loss, fatigue or loss of appetite and had no history of previous malignancies.

**Fig. 1:**
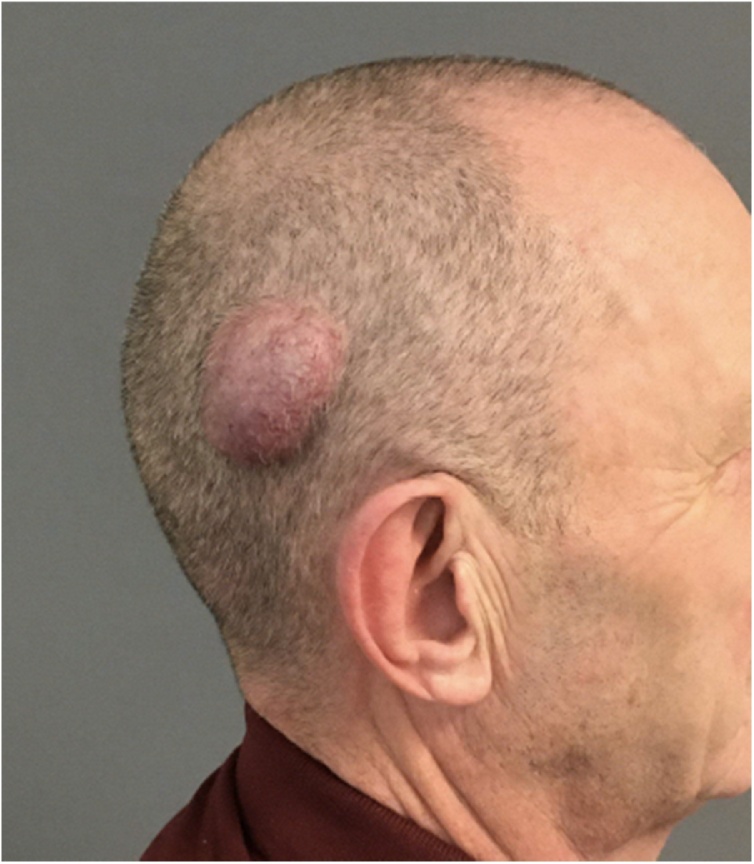
Clinical presentation of the tumor.

Clinical examination revealed a 4 × 5 cm, erythematous tumor in the right occipital region (Fig. 1). On palpation the tumor appeared pulsating, soft and non-adherent to the underlying bone. There were no palpable lymph nodes regionally. Differential diagnoses considered were other vascular tumors such as pyogenic granuloma, Kaposi sarcoma and angiosarcoma.

CT-angiography showed a richly vascularized, extracranial tumor with multiple large veins. The tumor was surgically excised under local anesthesia with a 5 mm margin and the defect reconstructed with a split skin graft.

**Fig. 2:**
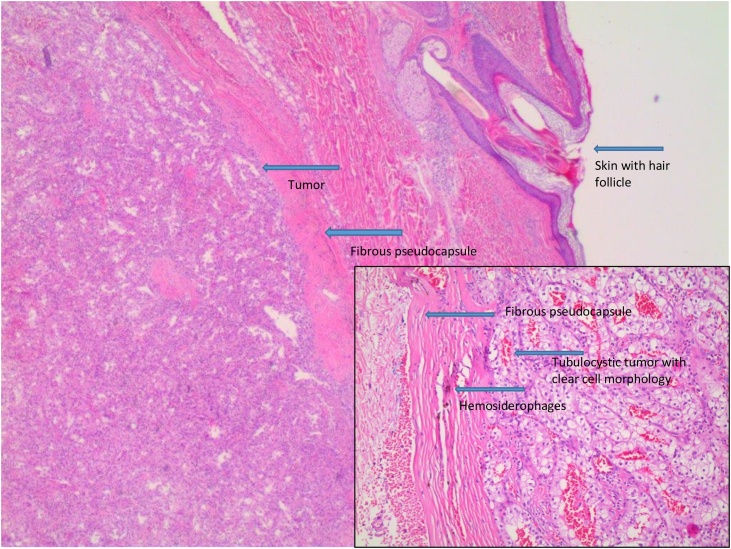
Skin with epidermis and dermis, tubulocystic tumor with clear cell morphology in dermis, Hematoxylin-Eosin X2,5.

**Fig. 3:**
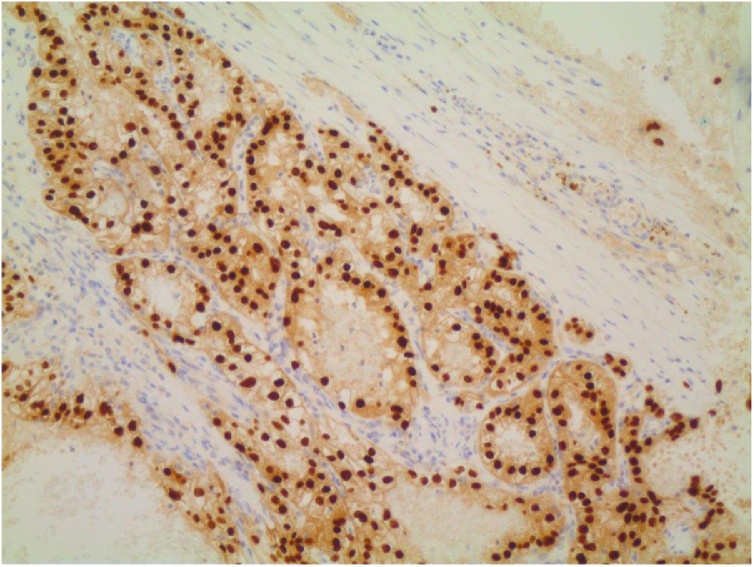
Immunohistochemical staining with PAX8, positive nuclear reaction, typical in kidney tumors. Magnification x20.

Histopathological examination revealed a well-defined mass in the deep dermis and subcutis, partly enclosed in a fibrous pseudocapsule. The process was formed by alveolar, tubular and tubulocystic formations of cells with clear cytoplasm and central, round nuclei (Figs. 2 and 3). Immunohistochemistry showed the lesion was positive for broad-spectrum cytokeratin, Vimentin and PAX8, and negative for S-100 and Leucocyte Common Antigen. The tumor was excised with radical margins and the morphology and immunophenotype were consistent with a metastasis from a clear cell renal cell carcinoma.

**Fig. 4:**
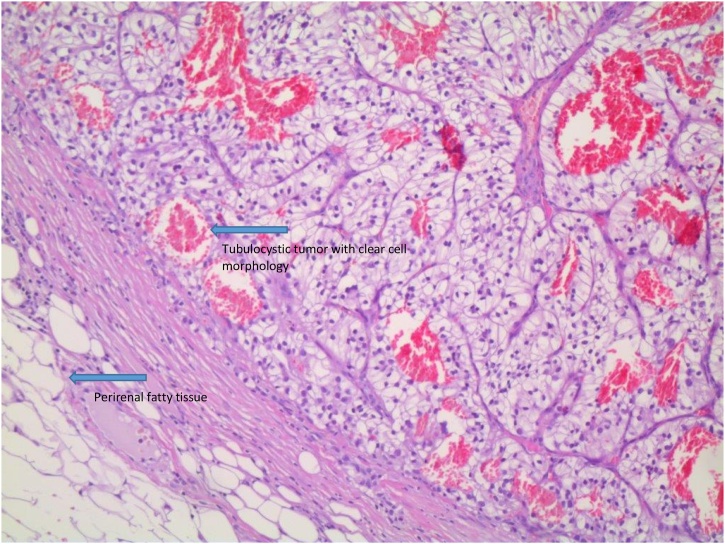
Kidney tumor with clear cell morphology, Hematoxylin-Eosin x10.

The patient was referred to the department of urology where a CT-scan and a PET-CT were obtained and revealed a 13 cm tumor in the left kidney, small lung metastases bilaterally and multiple suspicious mediastinal lymph nodes. The patient was discussed at a multidisciplinary team-conference and subsequently underwent a debulking laparoscopic nephrectomy, confirming a primary 10 cm clear cell renal cell carcinoma with vascular invasion (Fig. 4), pT3a. No systemic treatment was initiated. Follow-up CT-scan three months postoperatively showed that one of the lung metastases had grown 3 mm, but otherwise no progression in the disease.

## 3 Discussion

Cutaneous metastases from visceral malignancies develop in approximately 5–10% of cancer patients and most commonly originate from cancers in the breast, lung, colon, ovaries and malignant melanomas [4]. Skin metastases from RCC are unusual, but a known entity, that occur in about 3.4% of cases [5]. In most cases they appear in patients with a history of RCC, often years after nephrectomy. There are reports of skin metastasis being the first symptom of RCC, but it is considered rare [6,7].

RCC metastases develop through local invasion, lymphatic or haematogenous pathways. The richly vascularized structure of RCCs is thought to facilitate hematogenous spread and the development of distant metastases. Skin metastases can appear anywhere on the body but are most commonly seen on the face and scalp. The appearance of the metastases vary but are often nodular and described as purple or vascular [4]. In our case the patient presented with a pulsating tumor, a characteristic that has been frequently reported for metastatic skin lesions and may cause clinical confusion with pyogenic granuloma [8,9].

Skin metastasis is a sign of advanced disease and is in most cases associated with metastases in other regions [10]. The prognosis for patients with a metastasis to the skin is poor. More than 98% of patients lived less than 12 months after a cutaneous metastasis from a urological malignancy was found [5].

## 4 Conclusion

We present a case where a tumor on the scalp led to the diagnosis of a disseminated renal cell carcinoma. Skin metastasis from RCC is, although rare, an important differential diagnosis to consider in skin tumors with a vascular appearance, particularly in patients with a previous history of RCC.

## Funding

This research did not receive any specific grant from funding agencies in the public, commercial, or not-for-profit sectors.

## Ethical approval

This case report is exempt from ethical approval in our institution.

## Consent

Written consent was given by the patient for publication of this case report with accompanying images.

## Author contribution

Christina Krogerus: Concept and design, data collection and interpretation, writing the paper.

Matilda Svenning: Concept and design, data collection and interpretation.

Anette Pilt: Data collection and interpretation, review and editing.

Hannah Trøstrup: Data interpretation, review and editing.

## Registration of research studies

N/A.

## Guarantor

Christina Krogerus.

## Declaration of Competing Interest

The authors report no declarations of interest.
